# Supplementary material for: Ant identity determines the fungi richness and composition of a myrmecochorous seed
Source: PLoS One. 2024 Mar 7;19(3):e0293377. doi: 10.1371/journal.pone.0293377 (PMC10919741; doi:10.1371/journal.pone.0293377)
Supplement: S1 Table — “Morphotype Frequency” is the number of seeds contaminated by the fungal morphotypes. The “Indicator value” represents the morphotype association with a given group of seeds. (DOCX) [file pone.0293377.s001.docx]

**Supplementary Table**

**Ant identity determines the fungi richness and composition of a myrmecochorous seed**

Tiago V. Fernandes^1,2,3^ *, Otavio L. Fernandes^4^, Inácio J. M. T. Gomes^1,3^, Ricardo R. C. Solar^2^,

Ricardo I. Campos^3^.

^1^ Programa de Pós-Graduação em Biologia Animal, Universidade Federal dos Vales do Jequitinhonha e Mucuri. Diamantina, Brazil.

^2^ Programa de Pós-Graduação em Ecologia, Conservação e Manejo da Vida Silvestre. Universidade Federal de Minas Gerais. Instituto de Ciências Biológicas, Belo Horizonte, Brazil.

^3^ Universidade Federal de Viçosa, Departamento de Biologia Geral, Viçosa-MG, Brazil.

^4^ Universidade Federal de Viçosa, Departamento de Entomologia, Viçosa-MG, Brazil.

**Table 1:** List of fungi morphotypes found on seeds of *Mabea fistulifera* after manipulation by *Acromyrmex subterraneus* (80 seeds), *Atta sexdens* (80 seeds), or non-manipulated (control, 160 seeds). “Morphotype Frequency” is the number of seeds contaminated by the fungi morphotype. The “Indicator value” represents the morphotype association with a given group of seeds. Highlighted (*) morphotypes frequencies show significative Indicator value (p < 0.05) of a morphotype to one or a combination of two groups of seeds.

| **Morphotype family** | **Morphotype** | **Morphotype Frequency** | | | **Indicator value (%)** |
| --- | --- | --- | --- | --- | --- |
|  |  | ***Acromyrmex*** | ***Atta*** | **Control** |  |
| Aspergillaceae | *Aspergillus* sp.1 | 16 | 1 | 5 | 48% |
|  | *Aspergillus* sp.2 | 7 | 8 | 5 | 30% |
|  | *Aspergillus* sp.3 | 6 | 11 | 1 | 28% |
|  | *Penicillium* sp.1 | **19*** | 0 | 8 | 83% |
|  | *Penicillium* sp.2 | 3 | 1 | 6 | 21% |
|  | *Penicillium* sp.3 | 3 | 0 | 0 | 50% |
|  | *Penicillium* sp.4 | 0 | 1 | 0 | 25% |
| **Morphotype family** | **Morphotype** | **Morphotype Frequency** | | | **Indicator value (%)** |
|  |  | ***Acromyrmex*** | ***Atta*** | **Control** |  |
| Chaetomiaceae | *Chaetomium* sp. | 1 | 3 | 9 | 34% |
| Cladosporiaceae | *Cladosporium* sp. | 33 | 38 | 53 | 38% |
| Hypocreaceae | *Acremonium* sp. | 7 | 4 | 7 | 36% |
|  | *Gliocladium* sp.1 | 4 | 0 | 1 | 44% |
|  | *Gliocladium* sp.2 | 0 | 0 | 1 | 13% |
|  | *Trichoderma* sp.1 | **17*** | 5 | 6 | 68% |
|  | *Trichoderma* sp.2 | 0 | 2 | 0 | 50% |
| Nectriaceae | *Fusarium* sp.1 | **7*** | **8*** | 1 | 39% |
|  | *Fusarium* sp.2 | 0 | 3 | 9 | 30% |
|  | *Fusarium* sp.3 | 0 | 7 | 0 | 50% |
| Periconiaceae | *Periconia* sp. | 2 | 2 | 1 | 22% |
| *Incertal sedis* | *Trichothecium roseum* | **5*** | **8*** | 0 | 46% |
| N/I | Sterile mycelium sp.1 | 5 | 0 | 6 | 46% |
|  | Sterile mycelium sp.2 | 1 | 0 | **62*** | 84% |
|  | Sterile mycelium sp.3 | 1 | 0 | 12 | 53% |
|  | Sterile mycelium sp.4 | 4 | 0 | 2 | 14% |
|  | Sterile mycelium sp.5 | 0 | 3 | 3 | 33% |
